# Progress in Psychiatry

**Published:** 1900-06-09

**Authors:** 


### Progress in Psychiatry.

(*Continued from page 71.*)

**The Stigmata of Degeneracy.**—The indications of degeneracy which an individual may exhibit are termed the stigmata of degeneracy. The tendency to the development and perpetuation of nervous and mental diseases (neuropathic and psychopathic taint) must be regarded as but one branch of the tree of degeneracy, a branch having collateral relationships with other branches of the same tree. Such branches include the tendencies to tuberculosis and phthisis, to arthritic and allied conditions (rheumatism, gout, and probably migraine), and to other vices of nutrition which the organism may exhibit. The affections of the nervous system which arise from the neuropathic or psychopathic taint may manifest themselves as simple sensorial, kinæsthetic, or psychical troubles, or as diverse and complex combinations of these; yet they form a natural whole, constituting a vast family in which the various members are more or less closely united under the laws of heredity. The main characteristics of the degenerative taint are: Firstly, that it tends to be transmitted from parent to offspring (subject to the laws of heredity we have enunciated in a previous chapter); and, secondly, that it tends to produce extinction of those members of the stock in whom the degenerative taint shows itself most markedly. Thus, Morel has shown ("Traite des Degenerescences Humaines," 1858) that the transmission of the vesaniae, *i.e.*, the simple insanities like melancholia and mania, is scarcely prolonged in families beyond the fourth generation, and this is a fact which alone suffices for him to characterise the degeneracy of these families. The genealogical tables published in previous chapters (Vol. XXVII., p. 327, and pp. 70-71 supra) will on careful study be also found to support this view.

The stigmata of degeneracy are thus external indications of the taint within, and, according to circumstances, they may assume various forms. They should be distinguished from accidental deformities, such as intra-uterine amputation of limbs, or accidents sustained during parturition and infancy. A lengthy list may be published containing the various stigmata, but many of the items so included are at present of doubtful import. The following, however, are the chief and most important of them, arranged under the headings of anatomical stigmata and psychical stigmata:—

(*a*) **Anatomical or Physical Stigmata of Degeneracy.**—These include such conditions as microcephaly, hydrocephalus, marked plagio-cephaly or asymmetry of the skull in its right and left halves, marked facial asymmetry with or without difference of levels between the right and left orbits and ears, marked prognathism approximating to the simian type, strabismus, ears ad ansa, ears with marked development of the Darwinian tubercle and absence of the lobule as in apes, cleft palate and hare-lip, narrow and high-arched or deformed palate and dental arch, arrested development of legs with tendency to a feminine type of build in males, abnormalities of digits such as polydactylism and syndactylism, and abnormalities of the sexual organs such as pseudo-hermaphroditism, hypospadias, cryptorchidism, &c., and general conditions such as dwarfishness and gigantism.

(b) **Psychical or Functional Stigmata of Degeneracy.**—These include such conditions as tics and allied disorders of movement, stuttering, congenital criminal instincts, congenital or early sexual perversion, depraved appetite, such as eating of earth, hair, filth, &c., irresistible impulses to steal, drink, &c., and tendencies to suicide during childhood. Magnan, who has carefully analysed and studied the groups of physical and psychical stigmata, finds the latter group to be composed of two classes of phenomena, viz., obsessions and impulses. Obsessions are organised strong nervous discharges predominating from time to time in the sensorial or psychical centres of the brain, and accompanied by special emotional disturbances, while impulses are nervous discharges predominating in the psychical and kinæsthetic centres and tending to issue in acts and deeds. An impulse need not necessarily be preceded by an obsession; it may and frequently does occur independently of obsession. Great precocity of intellect, of aptitude for art, and for emotional development in certain directions (art, religiosity, hysteria, and eroticism) are suspicious indications of a neuropathic or psychopathic basis in children. (An excellent and concise account of the stigmata of degeneration is to be found in Féré's "La Famille Neuropathique," 1894, pages 252-309.)

While many of the foregoing stigmata have but a limited significance, yet a combination of two or more will be found to have a more marked diagnostic and prognostic value. Among normal men, who are in the world at large, and able to work and earn a living, about 2 per cent. have stigmata of degeneration, whereas among the neuropathic and psychopathic classes the proportion is vastly increased. It is especially among epileptics and those who are subjects of neurasthenia, profound hysteria, idiocy, imbecility and weak-mindedness, and paranoia in its various forms, that these signs are to be looked for and studied. Among lunatics, criminals, paranoiacs, and congenital imbeciles about 30 per cent. or more will be found to exhibit stigmata of degeneracy in unmistakable form. It is an unfortunate fact that, while some observers (especially Lombroso) have laid too great a stress on and drawn too wide and sweeping conclusions from some of the so-called stigmata of degeneracy, others have been too ready and willing to minimise or deny their significance altogether. Both extremes should be avoided by the student. Much valuable matter has been gathered and published in recent works which will enable individual observers to form their own conclusions. The works of Morel, Magnan, Féré, Lombroso, Marro,[31] Benedikt,[32] Krafft-Ebing,[33] Tarnowski,[34] Havelock Ellis,[35] and MacDonald[36] contain most of the facts and observations made on the subject. The tendency to prosecute these studies among the criminal, the lunatic, and degenerate classes is one of the best signs of an awakening of interest among medico-psychological, legal, and philanthropic circles as to the seriousness of the problem which confronts society as regards lunatics, criminals, and degenerates. The provision for their accommodation and treatment has taken the forms of workhouses, asylums, prisons, penitentiaries, reformatories, and industrial colonies. For certain special classes of degenerates Professor Morel, of Belgium, Inspector of Lunatic Asylums and State Prisons, has recommended special "Institutions for the Degenerate,"[37] a recommendation which possesses great weight and authority and which demands serious consideration.

[31] I caratteri dei Delinquenti, Turin. [32] Kraniometrie und Kephalometrie; and in Havelock Ellis' "Criminal," passim. [33] Grundzuge der Kriminal Psychologie, 1872; and Psychopathia Sexualis. [34] Archives de l'Anthropologie Criminelle, 1889, &c., passim. [35] The Criminal, London, 1890. [36] Criminology, New York. [37] Journal of Mental Science, Oct., 1894.

